# Mechanisms of *Legionella pneumophila*-induced interleukin-8 expression in human lung epithelial cells

**DOI:** 10.1186/1471-2180-7-102

**Published:** 2007-11-22

**Authors:** Hiromitsu Teruya, Futoshi Higa, Morikazu Akamine, Chie Ishikawa, Taeko Okudaira, Koh Tomimori, Naofumi Mukaida, Masao Tateyama, Klaus Heuner, Jiro Fujita, Naoki Mori

**Affiliations:** 1 Division of Molecular Virology and Oncology, Graduate School of Medicine, University of the Ryukyus, 207 Uehara, Nishihara, Okinawa 903-0215, Japan; 2 Division of Control and Prevention of Infectious Diseases, Graduate School of Medicine, University of the Ryukyus, 207 Uehara, Nishihara, Okinawa 903-0215, Japan; 3 Division of Child Health and Welfare, Faculty of Medicine, University of the Ryukyus, 207 Uehara, Nishihara, Okinawa 903-0215, Japan; 4 Division of Endocrinology and Metabolism, Faculty of Medicine, University of the Ryukyus, 207 Uehara, Nishihara, Okinawa 903-0215, Japan; 5 Division of Molecular Bioregulation, Cancer Research Institute, Kanazawa University, 13-1 Takara-machi, Kanazawa 920-0934, Japan; 6 Institute for Molecular Infection Biology, Universitat Wuerzburg, Roentgenring 11, 97070 Wuerzburg, Germany

## Abstract

**Background:**

*Legionella pneumophila *is a facultative intracellular bacterium, capable of replicating within the phagosomes of macrophages and monocytes, but little is known about its interaction with human lung epithelial cells. We investigated the effect of *L. pneumophila *on the expression of interleukin-8 (IL-8) in human A549 alveolar and NCI-H292 tracheal epithelial cell lines.

**Results:**

Infection of *L. pneumophila *strain, but not heat-killed strain, resulted in upregulation of IL-8. IL-8 mRNA expression was induced immediately after the infection and its signal became gradually stronger until 24 h after infection. On the other hand, IL-8 expression in A549 cells infected with *L. pneumophila *lacking a functional type IV secretion system was transient. The IL-8 expression was slightly induced at 16 h and increased at 24 h after infection with flagellin-deficient *Legionella*. Activation of the IL-8 promoter by *L. pneumophila *infection occurred through the action of nuclear factor-κB (NF-κB). Transfection of dominant negative mutants of NF-κB-inducing kinase, IκB kinase and IκB inhibited *L. pneumophila*-mediated activation of IL-8 promoter. Treatment with hsp90 inhibitor suppressed *L. pneumophila*-induced IL-8 mRNA due to deactivation of NF-κB.

**Conclusion:**

Collectively, these results suggest that *L. pneumophila *induces activation of NF-κB through an intracellular signaling pathway that involves NF-κB-inducing kinase and IκB kinase, leading to IL-8 gene transcription, and that hsp90 acts as a crucial regulator in *L. pneumophila*-induced IL-8 expression, presumably contributing to immune response in *L. pneumophila*. The presence of flagellin and a type IV secretion system are critical for *Legionella *to induce IL-8 expression in lung epithelial cells.

## Background

*Legionella pneumophila *causes atypical pneumonia, especially in patients with chronic pulmonary diseases and underlying immunosuppression, and in elderly people. Although more than 40 species of *Legionella *are known, the majority of human infections are caused by *L. pneumophila*, particularly serogroup 1 [1,2]. *L. pneumophila *is a gram-negative, facultative intracellular pathogen of amoeba in natural and man-made aquatic environments. Infection of humans occurs after inhalation of contaminated water aerosol droplets.

*L. pneumophila *can multiply within the mononuclear cells *in vivo *and *in vitro *and evades phagosome-lysosome fusion within these cells. It contains an array of important virulence factors including the Dot/Icm type IV secretion system, which is important for bacterial invasion and replication in the host cells [3]. Several *L. pneumophila *virulence factors that facilitate intracellular growth have been identified by analyzing infection of protozoans or immunocytes like macrophages [3,4]. However, it has been estimated that there are 28,000 type I pneumocytes, 1,400 type II pneumocytes and 50 alveolar macrophages per alveolus in an average human male [5,6]. Thus, *Legionella *and other pathogenic organisms, which cause respiratory infection, might well interact with the epithelial cells lining the space.

Lung epithelial cells constitute the first mechanical and immunological barrier against airborne pathogens and are important sources of cytokines in the lung [7,8]. Although *Legionella *efficiently infects and stimulates lung epithelial cells [9,10], the mechanisms of *L. pneumophila*-induced activation of cytokine genes in lung epithelial cells are mostly unknown. When the organisms are inhaled into the respiratory tract, the epithelial cells react to defend against the invaders and intra-alveolar exudation of neutrophils is observed. Interleukin-8 (IL-8) is a chemotactic factor and activator of neutrophils, basophils and T cells [11] and is involved in the early host response to pathogens [12,13]. In fact, serum IL-8 concentration is a potential marker of *Legionella *pneumonia [14]. Therefore, we analyzed the signaling pathways for IL-8 activation in human lung epithelial cells by *Legionella *infection, using human A549 alveolar and NCI-H292 tracheal epithelial cell lines.

## Results

### Multiplication of *L. pneumophila *in human lung epithelial cell lines

**Figure 1:**
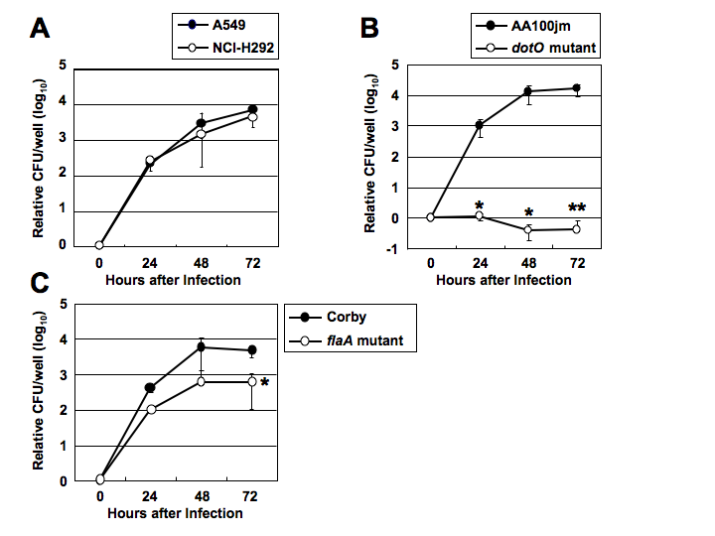
**Intracellular growth of the wild-type, *dotO *mutant and *flaA *mutant *L. pneumophila *strains in epithelial cells**. (A) A549 and NCI-H292 cells were infected with *L. pneumophila *AA100jm strain (MOI of 100). (B) A549 cells were infected with AA100jm and *dotO *mutant (MOI of 100). (C) A549 cells were infected with Corby and *flaA *mutant (MOI of 100). The number of bacteria at 0 h point is set as 1 and that at the indicated points is presented as the relative log_10 _CFU in cultures. Each data point represents the mean ± SD of triplicate cell cultures. *, *P *< 0.05; **, *P *< 0.01 (compared to cells infected with the wild-type strains).

We first examined intracellular growth of *L. pneumophila *strain AA100jm in A549 and NCI-H292 cells by 72-h continuous cultures. The colony forming unit (CFU) per well of AA100jm growing in A549 and NCI-H292 cell cultures began to increase after 24 h and then increased time-dependently (Figure 1A). However, the CFU of the avirulent mutant strain with a knockout in *dotO*, encoding a protein essential for the type IV secretion system, did not increase during the 72-h period (Figure 1B). Furthermore, the multiplication of *flaA *mutant was slightly inhibited in A549 cells compared with the wild-type Corby (Figure 1C). This observation suggests that flagellin plays a role in uptake into non-phagocytic cells.

### Infection of A549 and NCI-H292 cells by *L. pneumophila *induces IL-8 expression

**Figure 2:**
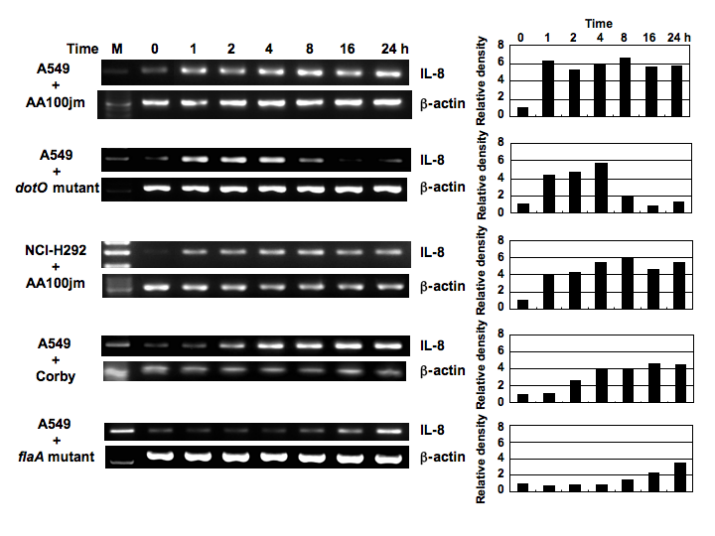
**Time course of *L. pneumophila*-induced IL-8 mRNA expression**. Total RNA was extracted from A549 and NCI-H292 cells infected with AA100jm, *dotO *mutant, Corby or *flaA *mutant (MOI of 100) for the indicated time intervals and used for RT-PCR. Histograms indicate the relative density data of IL-8 obtained by densitometric analysis of the bands normalized to β-actin.

**Figure 3:**
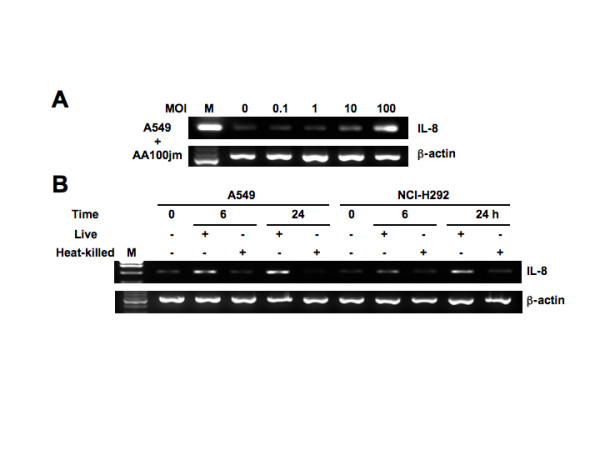
***L. pneumophila*-induced IL-8 mRNA expression in epithelial cells**. (A) *L. pneumophila *infection increases IL-8 mRNA expression in A549 cells in a dose-dependent manner. A549 cells were infected with varying concentrations of AA100jm, and the levels of IL-8 mRNA expression were examined by RT-PCR in cells harvested after 8 h. (B) Effect of heat-treatment of *L. pneumophila *on the ability to induce IL-8 mRNA expression. Expression of IL-8 mRNA in A549 and NCI-H292 cells treated with heat-killed AA100jm was observed at 6 and 24 h after infection. A549 and NCI-H292 cells were infected with the untreated AA100jm at an MOI of 100. β-actin expression served as controls. Representative results of three similar experiments in each panel are shown.

Monolayers of A549 and NCI-H292 cells were infected with *L. pneumophila *strain AA100jm for up to 24 h. Total cellular RNA was isolated from these cells at 1, 2, 4, 8, 16 and 24 h after the infection and IL-8 gene expression was analyzed by reverse transcription-polymerase chain reaction (RT-PCR). IL-8 mRNA expression increased immediately after the infection and its signal became gradually stronger in A549 and NCI-H292 cells until 24 h after infection (Figure 2). In another series of experiments, in which A549 cells were infected with AA100jm at different concentrations for 8 h (Figure 3A), AA100jm induced dose-dependent expression of IL-8 mRNA. Next, we examined the correlation between IL-8 expression level and the virulence of *L. pneumophila*. As shown in Figure 2, IL-8 mRNA expression was induced immediately after the infection and reached a peak level at 4 h, but became gradually weaker from 8 to 24 h after infection with the avirulent *dotO *mutant. Moreover, *Legionella *flagellin seemed to be involved in IL-8 expression, since a *flaA *knockout mutant, defective in flagellin production, failed to induce IL-8 mRNA immediately after the infection (Figure 2). However, its signal was induced by the *flaA *mutant at 16 h post-infection.

To determine the correlation between IL-8 expression levels and bacterial proteins of *L. pneumophila*, heat-killed AA100jm was used to infect A549 and NCI-H292 cells at a multiplicity of infection (MOI) of 100. IL-8 mRNA expression levels at 6 and 24 h in both cell lines infected with the heat-killed strain were significantly lower than those of cell lines infected with the live strain (Figure 3B). Furthermore, paraformaldehyde-fixed *L. pneumophila *showed no induction of epithelial IL-8 gene expression (data not shown).

### IL-8 production from A549 cells during infection with *L. pneumophila*

**Figure 4:**
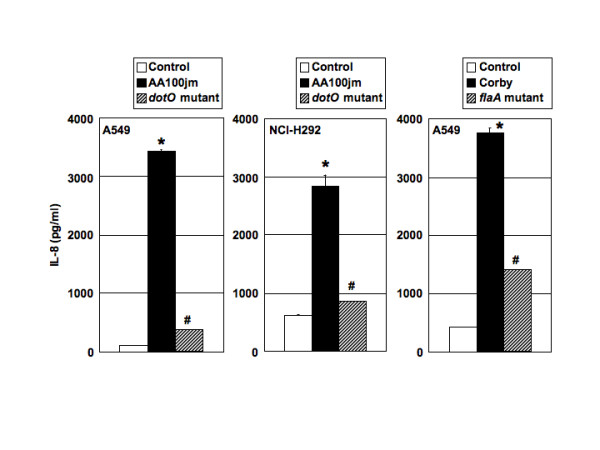
**Secretion of IL-8 into the supernatants of A549 and NCI-H292 cultures in response to either AA100jm or *dotO *mutant and either Corby or *flaA *mutant infection at 24 h**. A549 and NCI-H292 cells were infected with *L. pneumophila *at an MOI of 100. Data are mean ± SD of three experiments. *, *P *< 0.001 (compared to uninfected cells). #, *P *< 0.001 (compared to cells infected with the wild-type strains).

We used enzyme-linked immuno-sorbent assay (ELISA) to determine IL-8 protein levels in culture supernatants of A549 and NCI-H292 cells at 24 h after infection with either the parental strain AA100jm or *dotO *mutant strain and either the parental strain Corby or *flaA *mutant strain at an MOI of 100. *L. pneumophila *did not alter the cell viability within an MOI of 100 and time frame tested (data not shown). We found IL-8 induction by AA100jm and Corby. On the other hand, the production levels of IL-8 by A549 and NCI-H292 cells infected with the *dotO *and *flaA *mutant strains were significantly less than those of cells infected with the wild-type strains (Figure 4).

### *L. pneumophila *induces IL-8 gene transcription via a sequence spanning positions -133 to -50 of the IL-8 gene promoter

**Figure 5:**
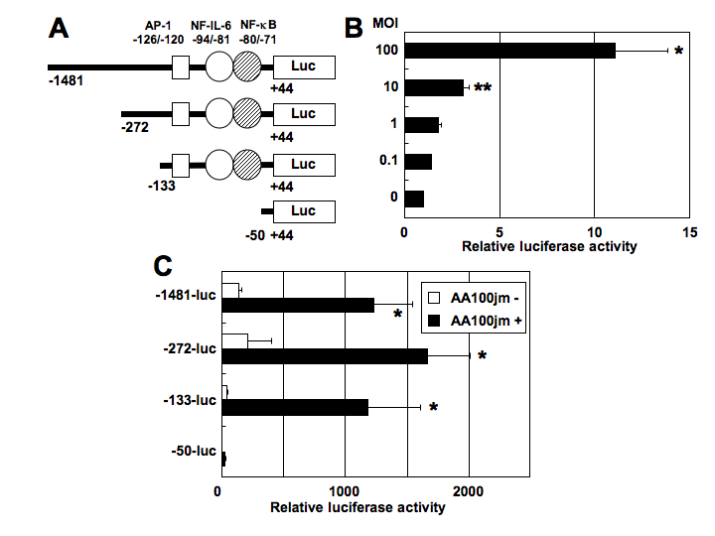
***L. pneumophila *infection activates IL-8 promoter in epithelial cells**. (A) Schematic representation of the IL-8 reporter constructs, demonstrating locations of several known binding sites for transcription factors. (B) *L. pneumophila *infection increases IL-8 promoter activity in a dose-dependent fashion. The -1481-luciferase construct (40 ng) containing IL-8 promoter spanning -1,481 to +44 bp was transfected into A549 cells, and the cells were subsequently infected with varying concentrations of *L. pneumophila *AA100jm for 48 h before luciferase assay. Luciferase activity is presented as a fold induction relative to the basal level measured in uninfected cells. (C) 5' deletion analysis of the IL-8 promoter. The indicated luciferase reporter constructs (40 ng) derived from the IL-8 promoter were transfected into A549 cells, and the cells were subsequently infected with AA100jm strain (MOI of 100) for 48 h. The activities are expressed relative to that of cells transfected with -50-luc without further treatment, which was defined as 1. Data are mean ± SD values of three independent experiments. *, *P *< 0.05; **, *P *< 0.01 (compared to uninfected cells).

To delineate the mechanism by which *L. pneumophila *induces IL-8 gene transcription, we identified *L. pneumophila*-responsive promoter elements in the IL-8 promoter. This was achieved by transfecting A549 cells with various plasmid constructs containing the luciferase reporter gene driven by the IL-8 promoter (Figure 5A). Twenty-four hours post-transfection, cells were infected with *L. pneumophila *strain AA100jm. The 5' region 1,481 bp full-length promoter was reproducibly activated by *L. pneumophila *infection in an MOI-dependent manner (Figure 5B). These results indicate that *L. pneumophila *induces IL-8 expression in A549 cells at transcriptional level. Next, we used a deletion analysis approach to identify essential promoter elements for transcriptional upregulation following a stimulus. High induction levels were observed with a reporter construct containing IL-8 5'-flanking sequence starting with position -1,481 to position -133. Deletion of sequences upstream of position -50 abolished inducibility by *L. pneumophila *infection (Figure 5C). The IL-8 gene fragment spanning positions -133 to -50 bp contains three prominent DNA-protein interaction sites for the transcription factors AP-1, NF-IL-6 and nuclear factor-κB (NF-κB) (Figure 5A). This maps the region from -133 to -50 bp as a *L. pneumophila*-responsive region, which is likely to contain individual *L. pneumophila*-responsive regulatory elements.

**Figure 6:**
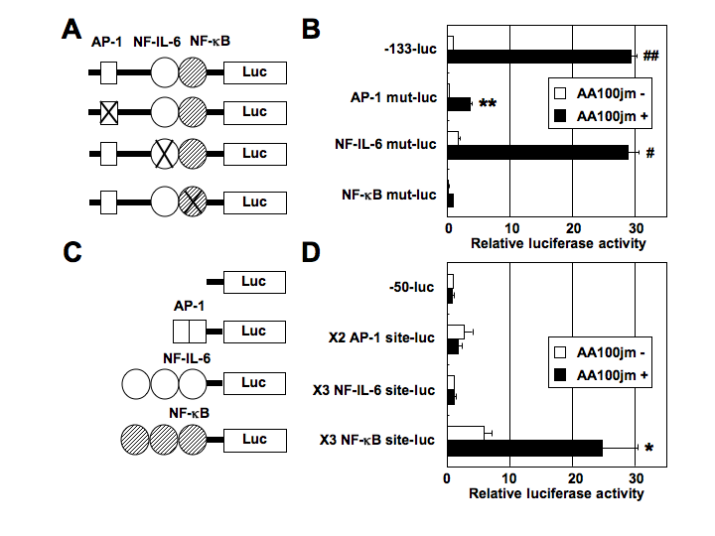
**The relative importance of AP-1, NF-IL-6 and NF-κB binding sites in the IL-8 promoter**. (A) Diagram of the constructs used in these experiments. The AP-1, NF-IL-6 or NF-κB site in the IL-8 promoter (-133 to +44 bp), linked to the luciferase gene, was mutated. (B) Mutational analysis of the *cis*-elements required for *L. pneumophila*-induced IL-8 promoter activity. The indicated wild-type and mutated plasmids (40 ng) were transfected into A549 cells, and the cells were subsequently infected with AA100jm strain (MOI of 100) for 48 h. The activities are expressed relative to that of cells transfected with -133-luc without further treatment, which was defined as 1. Data are mean ± SD values of three independent experiments. (C) Diagram of the constructs used in these experiments. (D) *L. pneumophila*-induced IL-8 gene expression is specific for the NF-κB region of the IL-8 gene. The activities are expressed relative to that of cells transfected with -50-luc without further treatment, which was defined as 1. Data are mean ± SD values of three independent experiments. *, *P *< 0.05; **, *P *< 0.005; #, *P *< 0.001; ##, *P *< 0.0005 (compared to uninfected cells).

Previous studies have shown that the AP-1 and NF-IL-6 sites act cooperatively with NF-κB to induce IL-8 gene transcription [15-17]. To identify the *cis*-acting elements in the -133 to -50 bp region of the IL-8 promoter, which served as a *L. pneumophila*-responsive regulatory element, we prepared and tested site-directed mutant constructs (Figure 6A). The results shown in Figure 6B indicate that mutation in the NF-κB site (NF-κB mut-luc) resulted in a significant reduction of *L. pneumophila*-induced IL-8 production. Mutation of the AP-1 site (AP-1 mut-luc) decreased basal activity but had little effect on *L. pneumophila*-induced luciferase activity (generated a 11.8-fold stimulation). However, mutation of the NF-IL-6 site (NF-IL-6 mut-luc) did not result in reduction of either basal activity or activity induced by *L. pneumophila *infection. These results indicate that activation of the IL-8 promoter in A549 cells in response to *L. pneumophila *infection requires an intact binding site for the NF-κB element.

### The NF-κB site of IL-8 gene can mediate *L. pneumophila*-induced gene activation

To further determine the exact site that is sufficient for *L. pneumophila*-induced IL-8 production, we used various constructs (Figure 6C). Each construct contained three tandemly repeated copies of one of the following elements: the NF-κB or NF-IL-6 site from the IL-8 gene or two copies of the AP-1 binding site from the IL-8 gene. Each was linked to the minimal IL-8 promoter spanning -50 to +44 bp and to the luciferase reporter gene. The construct × 3 NF-κB site-luc, which contains three tandem repeats of the NF-κB site from the IL-8 gene, generated a 4.2-fold stimulation by *L. pneumophila *infection. In contrast, constructs × 2 AP-1 site-luc and × 3 NF-IL-6 site-luc did not exhibit *L. pneumophila*-induced gene expression in A549 cells (Figure 6D). These results indicate that the NF-κB site of the IL-8 gene is important for activation of *L. pneumophila*-induced gene expression. In contrast, the AP-1 and NF-IL-6 sites, essential for IL-8 induction by various stimuli in other cell types, are not involved in this form of *L. pneumophila*-responsiveness.

### *L. pneumophila *infection induces a single predominant NF-κB binding complex, while AP-1 is constitutively expressed in A549 and NCI-H292 cells

**Figure 7:**
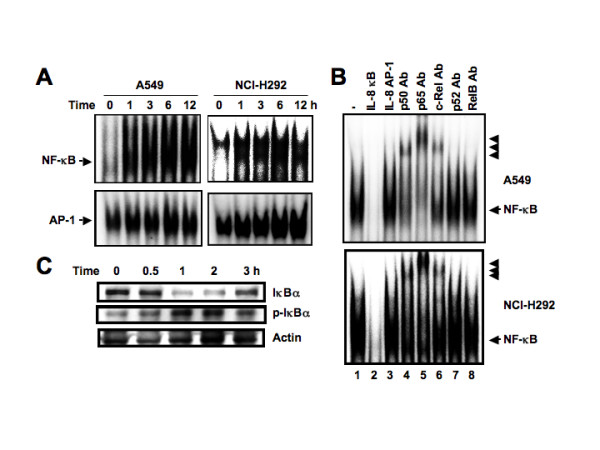
***L. pneumophila *infection induces NF-κB activation**. (A) Time course of NF-κB activation in A549 and NCI-H292 cells infected with *L. pneumophila*, evaluated by EMSA. Nuclear extracts from A549 and NCI-H292 cells, infected with AA100jm strain (MOI of 100), for the indicated time periods, were mixed with either NF-κB (upper panels) or AP-1 ^32^P-labeled probes (lower panels). Probes NF-κB and AP-1 are -83 to -68 bp and -130 to -116 bp fragments containing NF-κB and AP-1 binding sites of the IL-8 promoter, respectively. (B) Sequence specificity of NF-κB binding activity and characterization of NF-κB proteins that bound to the NF-κB binding site of the IL-8 gene. Competition assays were performed with nuclear extracts from A549 and NCI-H292 cells infected with AA100jm strain (MOI of 100) for 12 h. The competitor used was the NF-κB site of the IL-8 gene (lane 2). An unrelated AP-1 binding site was also used as a competitor (lane 3). Where indicated, 100-fold excess amounts of each specific competitor oligonucleotide (lanes 2 and 3) were added to the reaction mixture with labeled NF-κB probe. Specific bands are indicated by arrows. Supershift assay of NF-κB DNA-binding complexes in the same nuclear extracts was also performed. Gel shift assay reaction mixtures containing the same nuclear extracts and indicated antibodies were incubated for 45 min, and then ^32^P-labeled probes were added (lanes 4–8). Supershifted bands with anti-NF-κB p50, anti-NF-κB p65 and anti-c-Rel antibodies are indicated by arrowheads (lanes 4–6). (C) *L. pneumophila *infection leads to IκBα phosphorylation and degradation. A549 cells were infected with AA100jm strain (MOI of 100), for the indicated time periods. The cells were then lysed and analyzed by immunoblot with phospho-specific IκBα, IκBα and actin antibodies. Representative results of three similar experiments in each panel are shown.

To investigate the *trans*-acting factors involved in the activation of IL-8 by *L. pneumophila *infection, we performed electrophoretic mobility shift assay (EMSA) with a probe spanning positions -83 to -68 bp of the IL-8 promoter, which includes the NF-κB binding site at -80 to -71 bp (Figure 7A, upper panels). *L. pneumophila *infection induced a single predominant NF-κB binding complex. This binding was specifically competed by an excess of unlabeled NF-κB from the IL-8 gene (Figure 7B, lane 2) but not by an oligonucleotide containing AP-1 from the IL-8 gene (Figure 7B, lane 3). To further characterize the NF-κB complex, we performed supershift experiments using specific antisera. An antibody against NF-κB p50, p65 or c-Rel specifically shifted and diminished the formation of the complex (Figure 7B, lanes 4–6), while an antibody against p52 or RelB had no effect (Figure 7B, lanes 7 and 8). These data suggest that this DNA-protein complex represents a p50–p65 or p50-c-Rel heterodimer product. In contrast, AP-1 binding was constitutively present in nuclei from uninfected cultures, appearing as a single band on EMSA (Figure 7A, lower panels). This DNA-protein complex contained Fra-2 and JunD (data not shown). Nuclear extracts from *L. pneumophila*-infected A549 cells incubated with the AP-1 probe showed no increase in binding compared with uninfected extracts (Figure 7A, lower panels). Taken together, the results of cold competition and supershift assays indicate that *L. pneumophila*-induced proteins bind the NF-κB site but not the AP-1 site, which is in agreement with the functional data shown in Figure 6.

### Phosphorylation and degradation of IκBα by *L. pneumophila *infection

In resting cells, NF-κB proteins are predominantly sequestered in the cytoplasm by NF-κB inhibitory proteins IκBα and IκBβ. The activation of NF-κB requires phosphorylation of two conserved serine residues of IκBα (serines 32 and 36) and IκBβ (serines 19 and 23) within their N-terminal domain [18]. Phosphorylation leads to ubiquitination and 26S proteasome-mediated degradation of IκBs, thereby releasing NF-κB from the complex followed by its translocation to the nucleus to activate various genes [18]. To investigate whether NF-κB activation is mediated through alteration of phosphorylation of IκBα, A549 cells were infected with *L. pneumophila *and their protein extracts were checked for phosphorylated IκBα expression. *L. pneumophila *infection induced the phosphorylated IκBα. Kinetic analysis of *L. pneumophila*-induced degradation of IκBα in A549 cells revealed gradual replacement of IκBα levels (Figure 7C), suggesting that induction of phosphorylation of IκBα by *L. pneumophila *infection leads to degradation of IκBα protein.

### NF-κB signal is essential for induction of IL-8 expression by *L. pneumophila*

**Figure 8:**
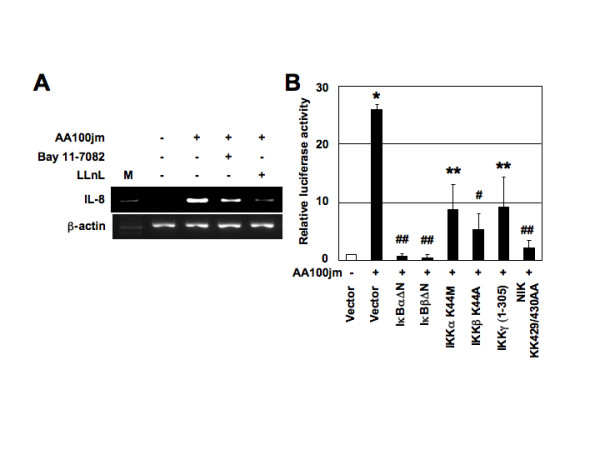
**NF-κB signal is essential for activation of IL-8 expression by *L. pneumophila***.(A) Bay 11-7082 and LLnL inhibit IL-8 mRNA expression induced by *L. pneumophila*. A549 cells were pretreated with Bay 11-7082 (20 μM) and LLnL (20 μM) for 2 h prior to AA100jm infection. They were subsequently infected with *L. pneumophila *for 6 h. IL-8 mRNA expression on harvested cells was analyzed by RT-PCR. Representative results of three similar experiments in each panel are shown. (B) Functional effects of IκBα, IκBβ and IKKγ dominant interfering mutants and kinase-deficient IKKα, IKKβ and NIK mutants on *L. pneumophila*-induced activation of the IL-8 promoter. A549 cells were transfected with 40 ng of -1481-luciferase construct and 2 μg of the indicated mutant plasmids or empty vector (pCMV4), and then infected with *L. pneumophila *(MOI of 100) for 48 h. Open bar represents luciferase activity of empty vector without *L. pneumophila *infection. All values were first calculated as a fold induction relative to the basal level measured in uninfected cells. Data are mean ± SD values of three independent experiments. *, *P *< 0.0005 (compared to uninfected cells). **, *P *< 0.05; #, *P *< 0.01;##, *P *< 0.005 (compared to cells transfected with empty vector with further *L. pneumophila *infection).

Since activation of the IL-8 promoter by *L. pneumophila *infection required activation of NF-κB, we blocked NF-κB activation with Bay 11-7082, an inhibitor of IκBα phosphorylation [19], or *N*-acetyl-Leu-Leu-norleucinaldehyde (LLnL), a proteasome inhibitor [20]. These studies served to demonstrate a link between NF-κB activation and upregulation of IL-8 expression in *L. pneumophila*-infected A549 cells. As shown in Figure 8A, Bay 11-7082 or LLnL markedly inhibited *L. pneumophila*-induced expression of IL-8 mRNA. We next examined whether *L. pneumophila*-mediated activation of IL-8 gene expression involves signal transduction components in NF-κB activation. A high-molecular-weight complex, IκB kinase (IKK) complex, which is composed of two catalytic subunits (IKKα and IKKβ) and a regulatory subunit (IKKγ), phosphorylates IκBs [18]. Members of the mitogen-activated protein kinase kinase kinase protein kinase family mediate physiological activation of IKK [21]. These kinases include NF-κB-inducing kinase (NIK) [22]. We tested the ability of dominant interfering mutants of IκBα, IκBβ and IKKγ, and kinase-deficient mutants of IKKα, IKKβ and NIK to inhibit *L. pneumophila*-mediated activation of the IL-8-driven reporter gene. Expression of these various inhibitory mutants abolished the induction of IL-8 promoter by *L. pneumophila *infection (Figure 8B). These data show that signaling components involved in the activation of NF-κB are necessary for *L. pneumophila *activation of IL-8 promoter.

### Inhibition of hsp90 reduces IL-8 expression induced by *L. pneumophila*

**Figure 9:**
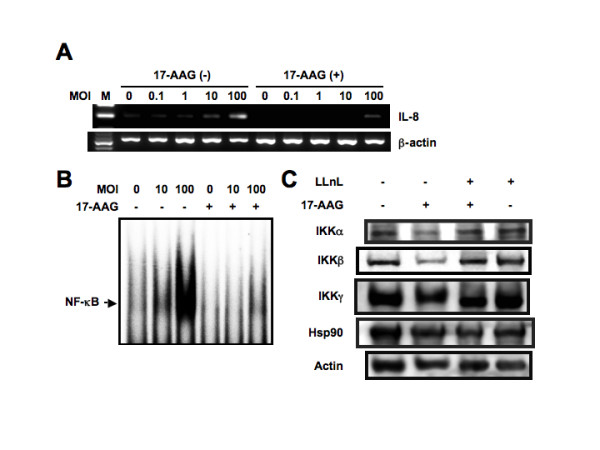
**Inhibitory effect of 17-AAG on *L. pneumophila*-induced IL-8 expression**.(A) A549 cells were incubated with 1 μM 17-AAG for 16 h prior to infection with varying concentrations of AA100jm strain for 6 h. RT-PCR was performed to check the changes of IL-8 mRNA expression after 17-AAG treatment in *L. pneumophila*-infected A549 cells. (B) Attenuation of *L. pneumophila*-induced NF-κB DNA binding by 17-AAG treatment. A549 cells were treated with (+) or without (-) 17-AAG for 16 h prior to infection with varying concentrations of *L. pneumophila *for 3 h. The nuclear extracts were isolated from A549 cells infected with *L. pneumophila *and incubated with ^32^P-labeled oligonucleotides corresponding to NF-κB. (C) hsp90 protects IKKα and IKKβ from proteasomal degradation. A549 cells either were pretreated with LLnL (20 μM) for 1 h, followed or not followed by addition of 17-AAG (1 μM) and incubation for 16 h, or were treated with 17-AAG for 16 h or left untreated as indicated. Whole cell extracts were immunoblotted with specific antibodies against each protein. Representative results of three similar experiments in each panel are shown.

The 90 kDa heat shock protein, hsp90 is a major molecular chaperone of the cell, and appears to have particular significance to cellular regulatory processes. Recent studies have revealed that most of client proteins of hsp90 are protein kinases or transcription factors that play important roles in cellular carcinogenesis [23,24]. Currently, several groups documented that hsp90 plays a critical role in inflammatory response and its inhibitor resulted in a reduced immune response as indicated by a decrease of proinflammatory mediator production [25-27]. Furthermore, a recent study indicated that IKKα and IKKβ are clients of hsp90 [28]. hsp90 has been found to associate stoichiometrically with the IKK complex by binding to the IKKα and IKKβ kinase domains [29]. As a possible mechanistic link between *L. pneumophila *infection and inflammation, we hypothesized the involvement of hsp90. To test this hypothesis, we investigated the effect of *L. pneumophila *infection on hsp90 and evaluated the effect of hsp90 inhibitor, 17-AAG, on *L. pneumophila*-induced IL-8 expression. A549 cells constitutively expressed hsp90 protein (Figure 9C), but *L. pneumophila *did not affect its expression (data not shown). Next, we analyzed whether hsp90 inhibitor could prevent *L. pneumophila*-induced IL-8 expression. For this purpose, A549 cells were pretreated with 17-allylamino-17-demethoxygeldanamycin (17-AAG) prior to *L. pneumophila *infection. The significant induction of IL-8 mRNA expression was completely inhibited by pretreatment of 17-AAG (Figure 9A). The finding suggests possible involvement of hsp90 in *L. pneumophila*-induced IL-8 signaling.

Because 17-AAG seemed to suppress *L. pneumophila*-induced IL-8 expression and NF-κB was identified as a critical regulator responsible for IL-8 transcription, we tested the direct influence of 17-AAG on *L. pneumophila*-induced transcriptional activity of NF-κB as a major transcriptional factor of IL-8 using by EMSA. Pretreatment with 17-AAG decreased the retardation of gel mobility through the inhibition of DNA binding activity of NF-κB complex, indicating repression of transcriptional activity of NF-κB (Figure 9B). Since a recent study indicated that IKKα and IKKβ are clients of hsp90 [28], we examined the effect of 17-AAG on expression of IKKα and IKKβ. Treatment of A549 cells with 17-AAG reduced the amounts of IKKα and IKKβ proteins but not those of IKKγ and hsp90 (Figure 9C). These results suggest that 17-AAG-mediated hsp90 inhibition depletes IKKα and IKKβ, resulting in inactivation of NF-κB.

Several hsp90 client proteins are degraded by the proteasome following hsp90 inhibition. To examine whether proteasomal degradation was responsible for decreased levels of client proteins after 17-AAG treatment, A549 cells were cultured in a medium containing 17-AAG and the proteasomal inhibitor LLnL. 17-AAG-mediated degradation of IKKα and IKKβ proteins was blocked by LLnL (Figure 9C). In contrast, IKKγ and hsp90 were not destabilized by 17-AAG, and LLnL did not change the levels of IKKγ and hsp90. The reversal of 17-AAG-induced degradation of the IKKα and IKKβ proteins by LLnL suggests that these proteins are subjects to ubiquitin-dependent turnover.

## Discussion

There is abundant information on *Legionella*, particularly *L. pneumophila*, infection and macrophages/monocytes. However, details about the interaction of *Legionella *with lung epithelial cells remain scarce. IL-8 is an inflammatory chemokine associated with immune-mediated pathology where it is involved in recruitment and activation of neutrophils and other immune cells [11]. The present study focused on IL-8 expression on human lung epithelial cells in *L. pneumophila *infection and discussed the possible transmission-signaling pathway of IL-8 induction.

Our results showed that *L. pneumophila *infection caused the expression and release of IL-8 from lung epithelial cells, which are the primary site of pulmonary infection [7]. The expression and production of IL-8 showed dose- and time-dependent patterns. However, the expression of IL-8 mRNA in A549 cells infected with the avirulent *dotO *mutant was transient and became weaker from 8 h post-infection than that in the cells infected with the wild-type strain. A previous study reported that infection of peritoneal macrophages of A/J mouse with *Legionella *resulted in production and release of IL-6, IL-1α, IL-1β and tumor necrosis factor-α, in culture supernatants; however, peritoneal macrophages never produced any cytokines when infected with an avirulent *L. pneumophila *strain [30]. These results were different from those of the present in which epithelial cells produced low levels of IL-8 when infected with the avirulent strain. This difference may be due to differences features of the two types of cells. Furthermore, we found that flagellin-deficient *Legionella *failed to induce IL-8 mRNA immediately after the infection, but its signal was induced at 16 h post-infection. Thus, in *L. pneumophila *infected epithelial cells, IL-8 upregulation seems to be controlled by two mechanisms: the first depends on substrate translocation via type IV secretion system and/or intracellular replication between 8 and 24 h while the other is bacterial growth-independent response induced immediately after the infection.

IL-8 gene transcription requires activation of the combination of NF-κB and either AP-1 or NF-IL-6, depending on the cell type [16,31-33]. These observations prompted us to investigate the contribution of these transcription factors to *L. pneumophila*-driven IL-8 gene transcription. In contrast to previous reports, we now report that *L. pneumophila*-induced IL-8 expression is independent of intact AP-1 and NF-IL-6 sites. These results suggest that different sets of nuclear transcription factors may be responsible for the regulation of IL-8 gene transcription in cell type- and stimulus-specific manners. Interestingly, AP-1 was the preferred transcription factor (over NF-IL-6) for cooperative interaction with NF-κB for IL-8 gene expression in respiratory syncytial virus-infected A549 cells [34]. However, our results demonstrated that activation of NF-κB, but not that of AP-1 or NF-IL-6, was indispensable for *L. pneumophila*-induced IL-8 gene transcription, suggesting that the molecular mechanism involved in IL-8 gene transcription differed, even within the same cell, depending on the employed stimulus.

The mammalian signaling pathways in epithelial cells that are triggered by *L. pneumophila *remain largely unknown. In the present study, we identified the cellular kinases NIK and IKKs as participants in NF-κB-dependent IL-8 induction by *L. pneumophila *in lung epithelial cells. Recently, it has been reported that *L. pneumophila *triggers nuclear localization of NF-κB in human and mouse macrophages in a Dot/Icm-dependent manner [35]. Thus, these studies and our results suggest that *L. pneumophila *activates IL-8 gene through Dot/Icm-dependent NF-κB activation. As concerns the expression and production of CCL20 that attracts immature dendritic cells and memory T cells, the similar results were obtained in *L. pneumophila*-infected A549 cells (data not shown). Since these data implicate a crucial involvement of NF-κB in *L. pneumophila*-provoked inflammation, NF-κB inhibitor may be a potentially useful therapeutic agent for Legionnaire's disease in addition to antibiotics.

A pathway exists for NF-κB activation via the cytosolic sensor Nod, which recognizes intracellularly localized peptidoglycan products [36]. There is evidence that peptidoglycan products are delivered across the plasma membrane by the *Helicobacter pylori *type IV secretion system, promoting Nod1-dependent NF-κB activation, so it is possible that Dot/Icm uses a similar strategy to activate NF-κB [37]. Furthermore, NIK participates in several Nod2-dependent cellular responses to peptidoglycan-derived structures [38]. Because Nod1 and Nod2 were expressed in A549 cells (data not shown), we are under investigation if activation of NF-κB by the Dot/Icm system is dependent on Nod1 and Nod2.

The *dotO *mutant could still induce IL-8 mRNA expression immediately after infection. Several host intracellular molecules are known to directly recognize bacteria and/or bacterial products, and trigger the appropriate inflammatory signaling pathway. NF-κB can be activated by engagement of toll-like receptors (TLRs) at the host cell surface in response to microbial molecules that are present on both pathogens and non-pathogens [39,40]. TLR5 is activated by flagellin protein, and in humans a common polymorphism in the TLR5 gene causes a deficiency in mediating signals from flagellin and increased susceptibility to Legionnaire's disease [41]. Flagellin-deficient *Legionella *failed to induce IL-8 mRNA expression immediately after infection. A549 cells used in the present study were observed to express TLR-5 by RT-PCR (data not shown). Induction of IL-8 may initially occur through other signaling pathways via TLR-5 engaged by surface-exposed *L. pneumophila *flagellum. After the completion of this work, Schmeck *et al*. reported that *L. pneumophila *induced NF-κB-dependent IL-8 release by lung epithelial cells [42]. However, they demonstrated that *L. pneumophila *srains induced flagellin-dependent but Dot/Icm-independent IL-8 release. Although N'Guessan *et al*. reported that IKK might contribute to *L. pneumophila*-induced-NF-κB signaling pathway, it is likely to be independent of Dot/Icm [43]. Why our results differ from them is unclear at this time. Because they did not remove bacteria after cells were inoculated with *L. pneumophila*, *dotA*-knock out mutant might induce IL-8 expression and NF-κB activation via flagellin of extracellular bacteria.

In addition, we documented, for the first time, the effect of hsp90 inhibitor, 17-AAG, on *L. pneumophila*-induced IL-8 expression and identified its molecular mechanism. 17-AAG inhibited IL-8 mRNA expression in *L. pneumophila*-infected lung epithelial cells. The finding may be due to inactivation of NF-κB signaling induced by *L. pneumophila *infection. hsp90 is a regulator of NF-κB signaling through its general involvement in IKK activation [28]. 17-AAG decreased IKK complex proteins, IKKα and IKKβ. The loss of IKK reduced NF-κB DNA-binding, resulting in reduced *L. pneumophila*-induced IL-8 mRNA expression.

## Conclusion

Our data provide important insights on the importance of hsp90 and NF-κB pathway in *L. pneumophila*-induced IL-8 production. It seems that recognition of extracellular pathogenic factors of *Legionella *such as flagella initially contributes to IL-8 induction and type IV secretion system Dot/Icm is essential for sustained IL-8 expression in epithelial cells infected with *L. pneumophila*.

## Methods

### Bacterial strain and cell culture

*L. pneumophila *serogroup 1 strain AA100jm [44] is a spontaneous streptomycin-resistant mutant of strain 130b, which is virulent in guinea pigs, macrophages and amoebae. The avirulent *dotO *mutant was constructed by random transposon mutagenesis, as described previously [44]. This mutation results in severe defects in intracellular growth and evasion of the endocytic pathway [45]. The Corby *flaA *mutant derived from the wild-type Corby is defective in flagellin [46]. *L. pneumophila *strains were grown at 35°C in a humidified incubator on either buffered charcoal-yeast extract-agar medium supplemented with α-ketoglutarate (BCYE-α) or in buffered yeast extract broth supplemented with α-ketoglutarate (BYE-α). Heat-killed bacteria were prepared by heating the bacterial suspension at 100°C for 1 h. Bacterial inactivation was achieved by paraformaldehyde (4%, 15 min followed by three washes in PBS) treatment. Both treated suspensions were confirmed to contain no viable bacteria by plating them on BCYE-α agar. Human A549, a type II alveolar epithelial cell line, and NCI-H292, a tracheal epithelial cell line, were maintained in RPMI 1640 containing 10% heat-inactivated FBS and were grown at 37°C in the presence of 5% CO_2_.

### Reagents

LLnL and Bay 11-7082 were purchased from Sigma-Aldrich (St. Louis, MO, USA) and Calbiochem (La Jolla, CA, USA), respectively. 17-AAG was purchased from Alomone Labs (Jerusalem, Israel). Rabbit polyclonal antibodies to IκBα and NF-κB subunits p65, p50, c-Rel, p52 and RelB were purchased from Santa Cruz Biotechnology (Santa Cruz, CA, USA). Mouse monoclonal antibodies to hsp90 and actin were purchased from BD Transduction Laboratories (San Jose, CA, USA) and NeoMarkers (Fremont, CA, USA), respectively. Mouse monoclonal antibody to phospho-IκBα (serines 32 and 36) and rabbit polyclonal antibodies to IKKα and IKKβ were purchased from Cell Signaling Technology (Beverly, MA, USA). Rabbit polyclonal antibody to IKKγ was purchased from Sigma-Aldrich.

### Infection of lung epithelial cells and intracellular growth kinetics experiments

Unless otherwise indicated, cells were inoculated with either the parental strain AA100jm or the mutant strain *dotO *and either the parental strain Corby or the mutant strain *flaA *at an MOI of 100 for 2 h. The extracellular fluid and bacteria were removed by washing three times with PBS, and cells were further incubated with fresh medium for the indicated time periods. In some experiments, heat-killed or paraformaldehyde-fixed bacteria were inoculated in the same manner. The intracellular growth assay of *L. pneumophila *in lung epithelial cells was performed in 24-well plates for 72 h. Cells were inoculated with either AA100jm or *dotO *mutant and either Corby or *flaA *mutant at an MOI of 100, followed by three times washing with PBS and 2 h gentamycin treatment (100 μg/ml) to kill any extracellular bacteria. The cells were washed three times again with PBS and were further incubated with fresh medium. The infected cells and supernatant in each well were harvested at the indicated time intervals by washing the wells three times with sterilized distilled water. These bacterial suspensions were diluted in sterilized water and plated in known volume onto BCYE-α agar. The numbers of CFU in infected monolayers were enumerated at the indicated time points after infection.

### IL-8 measurement

The IL-8 content in the culture supernatants was measured by ELISA (BioSource International, Camarillo, CA, USA). A549 cells were cultured in RPMI 1640 supplemented with 10% FBS in 24-well plates. Subconfluent monolayers of A549 cells were infected with *L. pneumophila *for 24 h. The supernatants were then collected after centrifugation and stored at -80°C until assayed for IL-8 by ELISA. The concentration of IL-8 was determined using a standard curve obtained with recombinant IL-8.

### RT-PCR

Total cellular RNA was extracted with TRIZOL reagent (Invitrogen, Carlsbad, CA, USA) according to the protocol provided by the manufacturer, and the amount of total RNA was determined by measuring the absorbance at 260 nm. First-strand cDNA was synthesized from 1 μg total cellular RNA in a 10-μl reaction volume using an RNA PCR kit (Takara Bio Inc., Otsu, Japan) with random primers. Thereafter, cDNA was amplified for 30 and 28 cycles for IL-8 and β-actin, respectively. The oligonucleotide primers used were as follows: for IL-8, sense, 5'-ATGACTTCCAAGCTGGCCGTG-3' and antisense, 5'-TTATGAATTCTCAGCCCTCTTCAAAAACTTCTC-3'; and for β-actin, sense, 5'-GTGGGGCGCCCCAGGCACCA-3' and antisense, 5'-CTCCTTAATGTCACGCACGATTTC-3'. Product sizes were 302 bp for IL-8 and 548 bp for β-actin. Cycling conditions were as follows: denaturing at 94°C for 30 s, annealing at 60°C for 30 s and extension at 72°C for 90 s. The PCR products were fractionated on 2% agarose gels and visualized by ethidium bromide staining. The photographs of the gels were scanned, and densitometry measurements of the scanned bands were performed using AlphaEase^® ^FC software (Alpha Innotech, San Leandro, CA, USA). Data were normalized to β-actin controls.

### Plasmids

The dominant negative mutants of IκBα, IκBαΔN, and IκBβ, IκBβΔN (provided by D. W. Ballard, Vanderbilt University School of Medicine, Nashville, TN, USA) are deletion mutants of IκBα and IκBβ lacking the N-terminal 36 amino acids and 23 amino acids, respectively [47,48]. The dominant negative mutants of IKKα, IKKα (K44M), and IKKβ, IKKβ (K44A), and dominant negative mutant of NIK, NIK (KK429/430AA) (provided by R. Geleziunas, Merk and Company Inc., West Point, PA, USA) and dominant negative mutant of IKKγ, IKKγ (1–305) (provided by K.-T. Jeang, National Institutes of Health, Bethesda, MD, USA), have been described previously [49,50]. Plasmids containing serial deletions of the 5'-flanking region of the IL-8 gene linked to luciferase expression vectors were constructed from a firefly luciferase expression vector [51]. Site-directed mutagenesis of the IL-8 AP-1, NF-IL-6 and NF-κB sites in the -133-luc plasmid was introduced, converting the AP-1 site TGACTCA (-126 to -120 bp) to TatCTCA, the NF-IL-6 site CAGTTGCAAATCGT (-94 to -81 bp) to agcTTGCAAATCGT and the NF-κB site GGAATTTCCT (-80 to -71 bp) to taAcTTTCCT (lower case letters indicate location of base changes). These constructs were designated as AP-1 site-mutated, NF-IL-6 site-mutated and NF-κB site-mutated plasmids, respectively. Two copies of the IL-8 AP-1 binding site, three copies of the NF-IL-6 site or three copies of the IL-8 NF-κB site were inserted upstream of the IL-8 enhancerless core promoter (-50 to +44 bp) linked to the firefly luciferase gene (-50-luc plasmid).

### Transfection and luciferase assay

A549 cells were transfected with 40 ng of appropriate reporter and 2 μg of effector plasmids along with 0.4 μg of phRL-TK, an internal control *renilla *luciferase expression vector (Promega, Madison, WI, USA), using Lipofectamine (Invitrogen). After 24 h, *L. pneumophila *was infected for 48 h. The cells were washed in PBS and lysed in reporter lysis buffer (Promega). Lysates were assayed for reporter gene activity with the Dual Luciferase Reporter Assay System (Promega). Relative luciferase amounts were normalized to equivalent *renilla *expression to control for transfection efficiency.

### Preparation of nuclear extracts and EMSA

NF-κB and AP-1 binding activities to NF-κB and AP-1 elements were examined by EMSA, as described previously [52]. In brief, 5 μg of nuclear extracts were preincubated in a binding buffer containing 1 μg poly(dI-dC)·poly(dI-dC) (Amersham Biosciences, Piscataway, NJ, USA), followed by addition of ^32^P-labeled oligonucleotide probes containing NF-κB and AP-1 elements from the IL-8 promoter (approximately 50,000 cpm). These mixtures were incubated for 15 min at room temperature. The DNA protein complexes were separated on a 4% polyacrylamide gel and visualized by autoradiography. To examine the specificity of the NF-κB element probe, we preincubated unlabeled competitor oligonucleotides with nuclear extracts for 15 min before incubation with probes. The probes or competitors used were prepared by annealing the sense and antisense synthetic oligonucleotides as follows: NF-κB of the IL-8 gene, 5'-gatcCGTGGAATTTCCTCTG-3'; and AP-1 of the IL-8 gene, 5'-gatcGTGATGACTCAGGTT-3'. Underlined sequences represent the NF-κB or AP-1 binding site. To identify NF-κB proteins in the DNA protein complex revealed by EMSA, we used antibodies specific for various NF-κB family proteins, including p65, p50, c-Rel, p52 and RelB to elicit a supershift DNA protein complex formation. These antibodies were incubated with the nuclear extracts for 45 min at room temperature before incubation with radiolabeled probes.

### Western blot analysis

Cells were lysed in a buffer containing 62.5 mM Tris-HCl (pH 6.8), 2% SDS, 10% glycerol, 6% 2-mercaptoethanol and 0.01% bromophenol blue. Equal amounts of protein (20 μg) were subjected to electrophoresis on SDS-polyacrylamide gels followed by transfer to a polyvinylidene difluoride membrane and probing sequentially with the specific antibodies. The bands were visualized with the enhanced chemiluminescence kit (Amersham Biosciences).

### Statistical analysis

Data were expressed as mean ± SD and analyzed by using the Student's *t*-test. A *P *value < 0.05 was considered to be significant.

## Authors' contributions

HT carried out the experiments and drafted the manuscript. MT and JF participated in the discussion on the study design. FH and MA contributed to the experimental concept and design and provided technical support. CI and TO participated in RT-PCR, EMSA, luciferase assay and Western blot analysis. KT carried out the IL-8 measurement. NMu carried out the generation of plasmids. KH undertook critical review of the manuscript. NMo conceived of the study, and participated in its design and coordination and helped to draft the manuscript. All authors read and approved the final manuscript.
