# Correction: mechanisms of *Legionella pneumophila*-induced interleukin-8 expression in human lung epithelial cells

**DOI:** 10.1186/1471-2180-11-136

**Published:** 2011-06-15

**Authors:** Hiromitsu Teruya, Futoshi Higa, Morikazu Akamine, Chie Ishikawa, Taeko Okudaira, Koh Tomimori, Naofumi Mukaida, Masao Tateyama, Klaus Heuner, Jiro Fujita, Naoki Mori

**Affiliations:** 1 Division of Molecular Virology and Oncology, Graduate School of Medicine, University of the Ryukyus, 207 Uehara, Nishihara, Okinawa 903-0215, Japan; 2 Division of Control and Prevention of Infectious Diseases, Graduate School of Medicine, University of the Ryukyus, 207 Uehara, Nishihara, Okinawa 903-0215, Japan; 3 Division of Child Health and Welfare, Faculty of Medicine, University of the Ryukyus, 207 Uehara, Nishihara, Okinawa 903-0215, Japan; 4 Division of Endocrinology and Metabolism, Faculty of Medicine, University of the Ryukyus, 207 Uehara, Nishihara, Okinawa 903-0215, Japan; 5 Division of Molecular Bioregulation, Cancer Research Institute, Kanazawa University, 13-1 Takara-machi, Kanazawa 920-0934, Japan; 6 Institute for Molecular Infection Biology, Universitat Wuerzburg, Roentgenring 11, 97070 Wuerzburg, Germany

## Correction

After the publication of this work [1], we became aware of the fact that β-actin control images in Figures two (*dotO *mutant), three A, eight A and nine A (figures 1, 2, 3 and 4 in this manuscript, respectively) were duplicated. The last author, Naoki Mori takes full responsibility for these errors in the original article. We repeated the experiments, and all the Figures mentioned above were deleted and new data substituted. The conclusions from the figures are not altered in any way. We regret any inconvenience that this inaccuracy in the original data might have caused.

**Figure 1:**
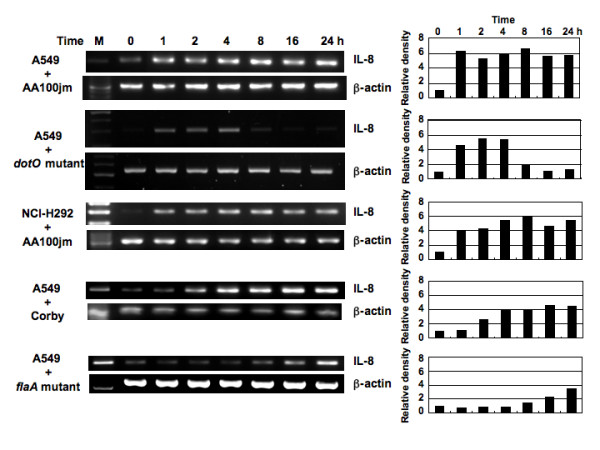
**Figure two - Time course of *L. pneumophila*-induced IL-8 mRNA expression**. Total RNA was extracted from A549 and NCI-H292 cells infected with AA100jm, *dotO *mutant, Corby or *flaA *mutant (MOI of 100) for the indicated time intervals and used for RT-PCR. Histograms indicate the relative density data of IL-8 obtained by densitometric analysis of the bands normalized to β-actin.

**Figure 2:**
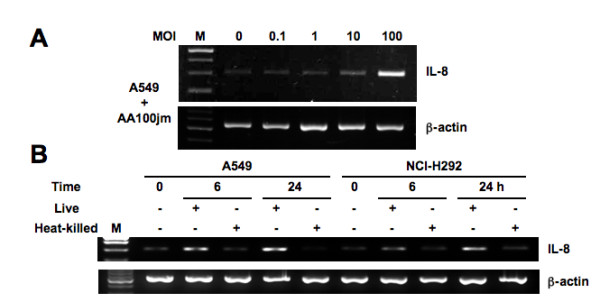
**Figure three - *L. pneumophila*-induced IL-8 mRNA expression in epithelial cells**. (A) *L. pneumophila *infection increases IL-8 mRNA expression in A549 cells in a dose-dependent manner. A549 cells were infected with varying concentrations of AA100jm, and the levels of IL-8 mRNA expression were examined by RT-PCR in cells harvested after 8 h. (B) Effect of heat-treatment of *L. pneumophila *on the ability to induce IL-8 mRNA expression. Expression of IL-8 mRNA in A549 and NCI-H292 cells treated with heat-killed AA100jm was observed at 6 and 24 h after infection. A549 and NCI-H292 cells were infected with the untreated AA100jm at an MOI of 100. β-actin expression served as controls. Representative results of three similar experiments in each panel are shown.

**Figure 3:**
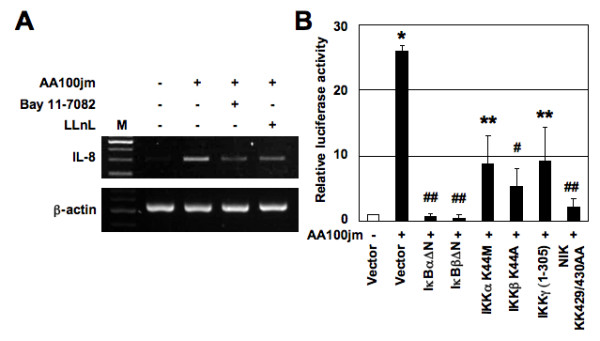
**Figure eight - NF-κB signal is essential for activation of IL-8 expression by *L. pneumophila***. (A) Bay 11-7082 and LLnL inhibit IL-8 mRNA expression induced by *L. pneumophila*. A549 cells were pretreated with Bay 11-7082 (20 μM) and LLnL (20 μM) for 2 h prior to AA100jm infection. They were subsequently infected with *L. pneumophila *for 6 h. IL-8 mRNA expression on harvested cells was analyzed by RT-PCR. Representative results of three similar experiments in each panel are shown. (B) Functional effects of IκBα, IκBβ and IKKγ dominant interfering mutants and kinase-deficient IKKα, IKKβ and NIK mutants on *L. pneumophila*-induced activation of the IL-8 promoter. A549 cells were transfected with 40 ng of -1481-luciferase construct and 2 μg of the indicated mutant plasmids or empty vector (pCMV4), and then infected with *L. pneumophila *(MOI of 100) for 48 h. Open bar represents luciferase activity of empty vector without *L. pneumophila *infection. All values were first calculated as a fold induction relative to the basal level measured in uninfected cells. Data are mean ± SD values of three independent experiments. *, *P *< 0.0005 (compared to uninfected cells). **, *P *< 0.05; #, *P *< 0.01;##, *P *< 0.005 (compared to cells transfected with empty vector with further *L. pneumophila *infection).

**Figure 4:**
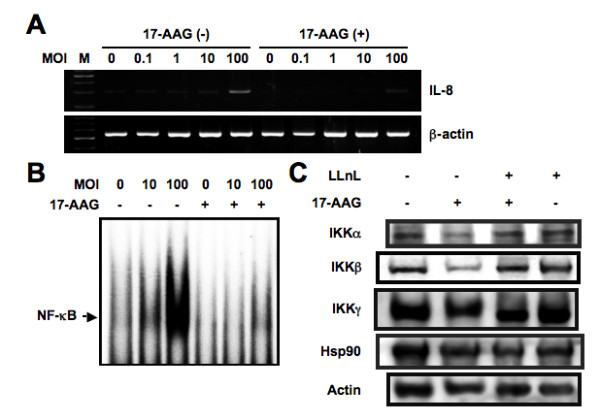
**Figure nine - Inhibitory effect of 17-AAG on *L. pneumophila*-induced IL-8 expression**. (A) A549 cells were incubated with 1 μM 17-AAG for 16 h prior to infection with varying concentrations of AA100jm strain for 6 h. RT-PCR was performed to check the changes of IL-8 mRNA expression after 17-AAG treatment in *L. pneumophila*-infected A549 cells. (B) Attenuation of *L. pneumophila*-induced NF-κB DNA binding by 17-AAG treatment. A549 cells were treated with (+) or without (-) 17-AAG for 16 h prior to infection with varying concentrations of *L. pneumophila *for 3 h. The nuclear extracts were isolated from A549 cells infected with *L. pneumophila *and incubated with ^32^P-labeled oligonucleotides corresponding to NF-κB. (C) hsp90 protects IKKα and IKKβ from proteasomal degradation. A549 cells either were pretreated with LLnL (20 μM) for 1 h, followed or not followed by addition of 17-AAG (1 μM) and incubation for 16 h, or were treated with 17-AAG for 16 h or left untreated as indicated. Whole cell extracts were immunoblotted with specific antibodies against each protein. Representative results of three similar experiments in each panel are shown.
